# Comprehensive Review of Spinal Neurenteric Cysts with a Focus on Histopathological Findings

**DOI:** 10.7759/cureus.3379

**Published:** 2018-09-28

**Authors:** Woong Kee Baek, Stefan Lachkar, Joe Iwanaga, Rod J Oskouian, Marios Loukas, W. Jerry Oakes, R. Shane Tubbs

**Affiliations:** 1 Anatomy, St. George's University, St. George, GRD; 2 Anatomy, Seattle Science Foundation, Seattle, USA; 3 Medical Education and Simulation, Seattle Science Foundation, Seattle, USA; 4 Neurosurgery, Swedish Neuroscience Institute, Seattle, USA; 5 Anatomy, St. George's University, St. George's, GRD; 6 Neurosurgery, Children's of Alabama, Birmingham, USA; 7 Neurosurgery, Seattle Science Foundation, Seattle, USA

**Keywords:** neurenteric cyst, intraspinal cyst, intraspinal endodermal cyst

## Abstract

Among the occult spinal dysraphisms, neurenteric cysts (NECs) are rare and are thought to arise due to a failure of the separation of the primitive endoderm and ectoderm. Patients experience various neurological symptoms depending on the location of the lesion. As the epithelial morphology of NECs share similarities with other intracranial and intraspinal cystic growths, the definitive diagnosis of NEC can be made after a histochemical analysis with endodermal markers. Complete resection is associated with the lowest disease recurrence rate.

## Introduction and background

Neurenteric cysts (NECs) are so rare that the various manifestations and different phenotypical associations of NECs render each case report and case series unique. This review examines the general anatomy and embryology of spinal NECs and emphasizes the related histopathology.

## Review

Anatomy

Intraspinal NECs make up 0.7%–1.3% of all spinal tumors and are found in males more often than females [1-2]. The population affected falls in the age group between neonate and early 70s with a higher incidence in children and young adults [3-4]. Depending on the location of the lesion, a spinal NEC can be described as intra- or extradural and intra- or extramedullary. The most common types are intradural and extramedullary lesions; they account for between 78% and 90% of the reported spinal NECs while intramedullary and extradural lesions account for the remaining cases [5-6]. Spinal NECs are predominantly found in the ventral to the ventro-lateral aspect of the spinal cord. The cervical vertebrae are the most common levels of its occurrence, followed by the thoracic and lumbosacral spine [2,7-8]. This predilection tendency may vary depending on the location of the NECs in relation to the spinal cord as isolated intramedullary subsets of this benign tumor are more frequent in the thoracic level [9].

The cystic growth can interfere with the local anatomy of the central nervous system. Patients experience a spectrum of symptoms from focal neurological deficits such as pain, paresthesia, motor deficits, progressive weakness, hyperreflexia, urinary incontinence, and tethered cord syndrome to more generalized symptoms such as paraplegia and quadriplegia [4-6,10-11]. The clinical presentations are related to the level of the lesion as the cystic mass can compress, flatten, and widen the affected level of the spinal cord [4,10]. Associated bone deformities include hemivertebrae, scoliosis, single- or multiple-level spina bifida, and Klippel–Feil syndrome [1,4,6,11]. Rauzzino et al. emphasized that associated cutaneous manifestations can also occur in patients with spinal NECs. These include hairy patches, pedunculated growth, capillary hemangioma, and subcutaneous masses [6]. However, a clear pattern of association between a specific vertebral or cutaneous deformity and spinal NECs has not been identified.

Embryology

The anatomical presentation and the histopathological illustration are closely related to the embryogenic defect associated with the condition. During the third week of gestation, the gastrulation takes place and embryonic trilaminar germ layers are formed. Cell proliferations followed by the proper migrations of different cell lineages determine the morphology and the primitive axis of the body. Mesodermal cells migrate to give rise to the notochord around which the paraxial mesoderm aggregates. The amniotic sac and yolk sac cavities are temporarily connected via the neurenteric canal, which later obliterates upon the development of the notochord. The ectoderm developing from the epiblast later gives rise to various parts of the nervous system. The endoderm, which is destined to line the gastrointestinal system and some part of the respiratory tract, replaces the hypoblast. 

**Figure 1:**
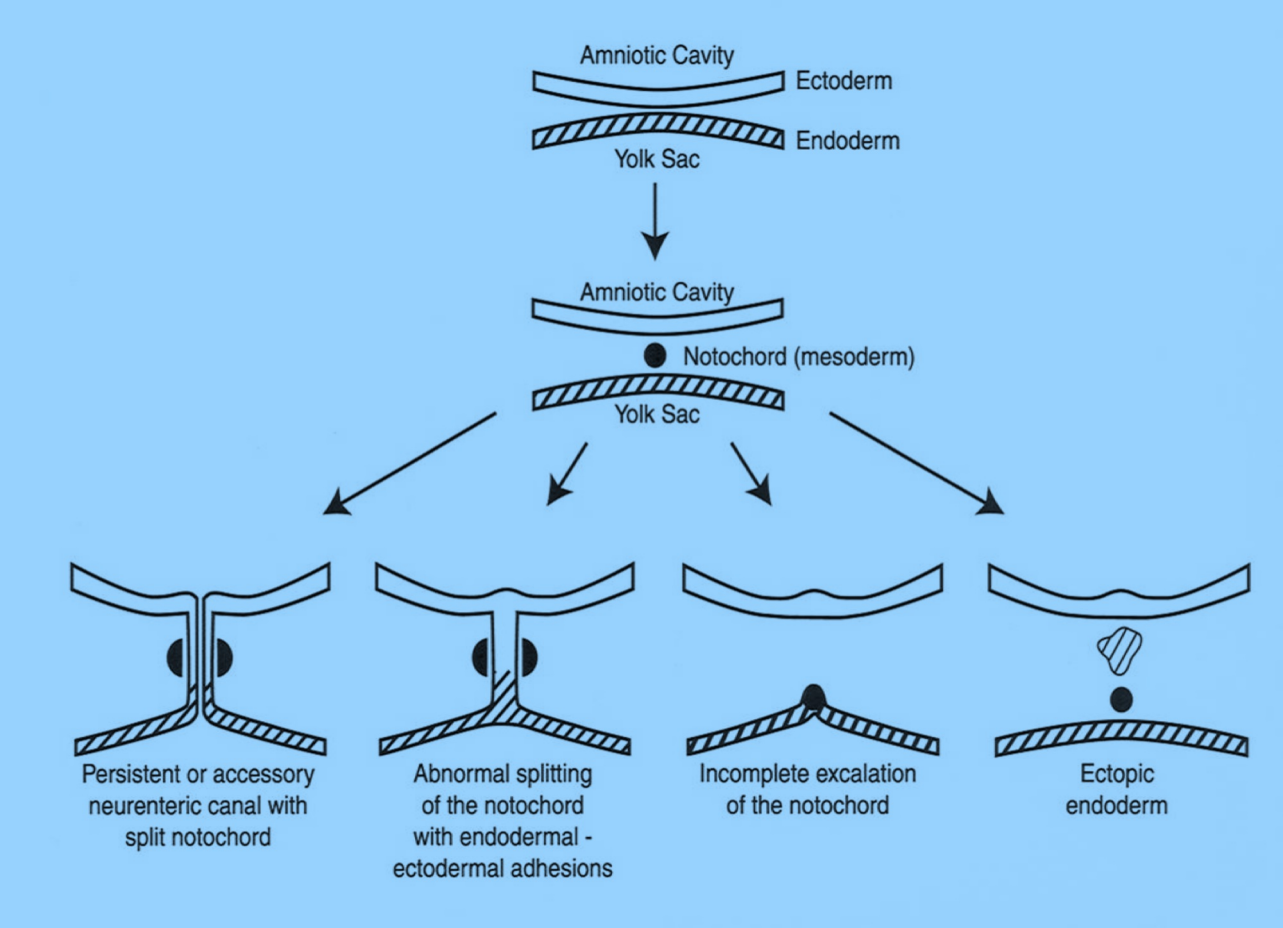
Schematic drawing of the proposed dysembryology resulting in various neurenteric cysts of the spine.

**Figure 2:**
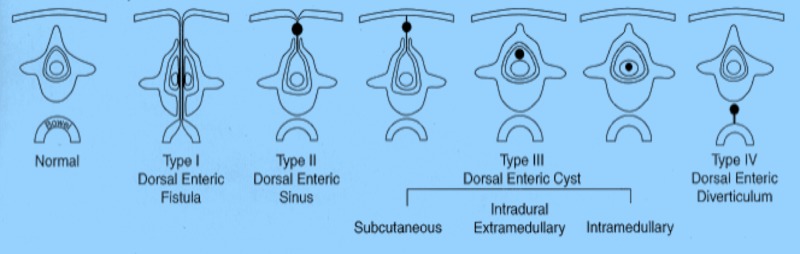
Enteric remnants and one of their classifications of these (Bentley and Smith Classification).

When the primitive endodermal cells fail to separate from the ectodermal counterparts, NECs are thought to be formed (Figures 1-2). Their walls are lined with well-differentiated cells of endodermal origin. The exact mechanism of pathogenesis behind this defect is not fully understood. Several postulates exist, and they can be grouped into five different mechanisms: (1) splitting of the notochord by endodermal tissue or a diverticulum [11-12], (2) adhesion between the notochord and the endodermal tissue, (3) failure of the obliteration of the neurenteric canal, (4) “incomplete escalation” of the notochord [13], and (5) abnormal notochord development secondary to incomplete ectoderm–endoderm separation [14].

Histology

**Figure 3:**
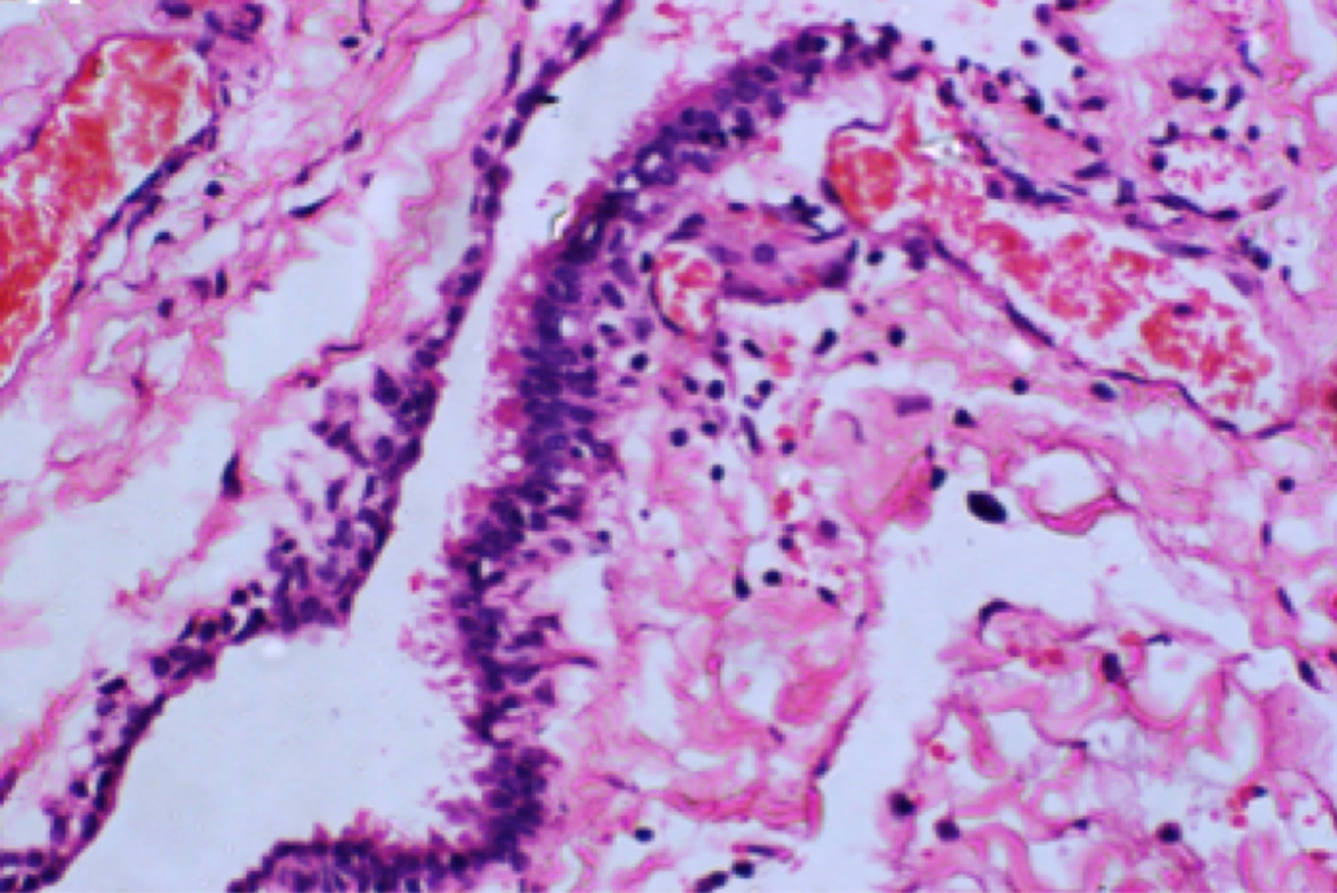
Histological section of spinal neurenteric cysts noting the typical epithelial lining seen with these pathological entities.

NECs are lined by cuboidal or columnar cells. The epithelial layer exhibits features of the gastrointestinal or respiratory lining with or without cilia, mucous glands, and/or goblet cells (Figure 3) [3-4,6,9-10,15]. These cysts can also be associated with the presence of other connective tissue components. Based on the epithelial integration to the basement membrane and/or the presence of other complex cellular types, Wilkins and Odum have proposed three different histopathological categories of NECs. Type A exhibits the simplest organization with a single layer of cuboidal to columnar cells that are associated with the basement membrane. These cells may or may not present with apical cilia. Type B has the features of type A in the presence of other connective tissue components such as glandular, cartilaginous, or lymphatic cellular histology. Type C is characterized by the cyst being lined by the cellular component of type A in the presence of glial and ependymal cells [16]. It is not clear under what circumstances the development of one cyst type is preferred over another.

A series of microscopic cellular analyses (Table 1) is required to differentiate NECs from other differential diagnoses of intraspinal cystic masses such as ependymal, epidermoid, or dermoid cysts. Differentiating neurenteric cysts solely on the basis of light microscopy from other intramedullary or intradural growths is also challenging [17-18]. For instance, intradural ependymal cysts exhibit similar cellular morphology with a ciliated simple cuboidal to columnar epithelium. However, these types of lesions are reactive with glial fibrillary acidic protein (GFAP) and S-100 protein along with other specific immunochemical markers for neuroglial cells [18-19]. Although the NEC is conventionally known to stain negative with GFAP, which is a neuroectodermal marker [1,20], focal positive reactivities are unusually observed in some cases [17].

**Table 1:** Histopathological features of neurenteric cysts *: generally negative. Unusual focal positivity has been reported **: stained positive only in the presence of goblet cells EMA: epithelial membrane antigen; AE1: anti-pan-cytokeratin antibodies; AE3: anti-pan-cytokeratin antibodies; hCG: human chorionic gonadotropin; PAP: placental alkaline phosphatase; CD31: cluster of differentiation 31; CK7: cytokeratin 7; CK20: cytokeratin 20; GFAP: glial fibrillary acidic protein; PAS: periodic acid Schiff; CAM 5.2: cell adhesion molecule; CEA: carcinoembryonic antigen; CA19.9: carbohydrate antigen; NSE: neuron-specific enolase; CDX2: caudal-type homeobox 2; MUC2: mucin 2; MUC5A: mucin 5AC; TTF-I: thyroid transcription factor-I; Ki67: nuclear antigen

	Neurenteric cyst
Associated connective tissue	Type A: associated with basement membrane [16] and loose connective tissue
	Type B: type A + glandular, lymphatic, nervous or smooth muscle component
	Type C: type A + ependymal cells, glial cells
Reactivity to immunochemistry	
PAS	+ [17,20-21]
Mucicarmin	+ [17,21]
Cytokeratin	+
CAM5.2	
AE1	+ [22]
AE3	+ [22]
CK7	+ [17,20,23]
CK20	+ [17,23]
CEA	+ [20-21]
CA19.9	
EMA	+ [21,23]
Vimentin	+ [23]
Ki67	low proliferative rate [21-22]
GFAP	- [22]*
S100 protein	- [21]
NSE	-
Transthyretin	-
CDX2	- [23]
MUC2	- [23]
MUC5A	+ [23] **
TTF-I	- [23]
hCG	- [23]
CD31	- [23]
PAP	- [23]

NECs, with the abundance of goblet cells and mucin-secreting cells, stain positive with periodic acidic Schiff and mucicarmine [3,20]. It is also known to be reactive with carcinoembryonic antigen (CEA), epithelial membrane antigen (EMA), cytokeratin 7 (CK7), and cytokeratin 20 (CK20) [17,20]. CK7 is known to be abundant in the lining of the foregut such as the pancreaticobiliary tree. CK20 is widely expressed by the epithelial lining of the small intestine and of the colon [21]. These markers confirm the primitive endodermal origin of NEC [17,20,23].

**Figure 4:**
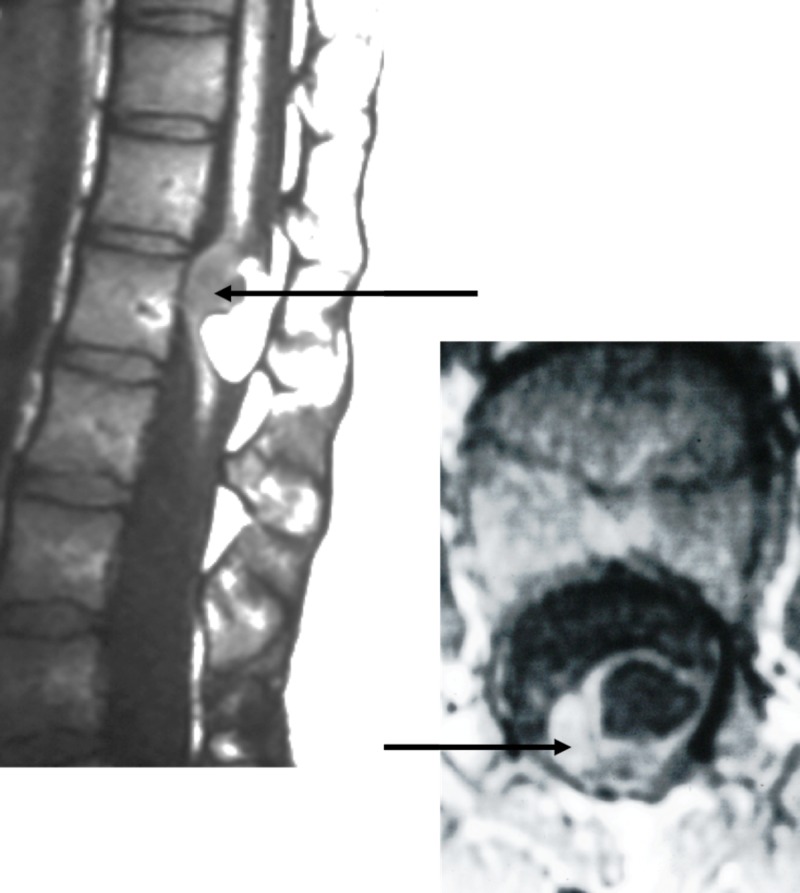
Left: Sagittal T1-weighted magnetic resonance imaging of the patient found to harbor a spinal neurenteric cyst (NEC) (arrow). Also note the dorsal lipoma posterior to the NEC; Right: Axial T2-weighted MRI of this patient depicting the NEC (arrow).

This cystic growth can also be associated with dystrophic calcification [9,17]. An intracranial and an isolated intramedullary neurenteric cyst case report suggested that an NEC can be considered as a differential diagnosis in the pathologies leading to intramedullary calcification [9]. The inner aspect of the neurenteric cystic cavity is filled with fluid. The physical appearance and the viscosity of these liquids may vary. Milky appearance [4], yellow or brown colored fluid [24] with water-like [10-11], mucous [18], “jelly-like” [3], or viscous consistencies are reported [11]. Molecular analysis of the fluid revealed a high protein concentration of variable degree with lipid substances. Magnetic resonance imaging (MRI) is the imaging modality to visualize spinal NECs (Figure 4), while computed tomography (CT) can be employed to depict the presence of associated dysraphisms. Due to the variations of the cystic content, the imaging intensity pattern on MRIs may be inconsistent. While the majority of the reported cases exhibit isointensity to hyperintensity signaling relative to the cerebrospinal fluid, on T1- and T2-weighted images, all spectrums of variations were reported [4,11,16]. The use of a fluid-attenuated inversion recovery (FLAIR) sequence MRI revealed mostly hyperintensity patterns [4,16]. A pattern of “rim enhancement” was detected in patients as a sign of chronic inflammation [4,25]. A recent study also had reported the presence of a solid nodular mass with melanin deposition [25].

Surgery

**Figure 5:**
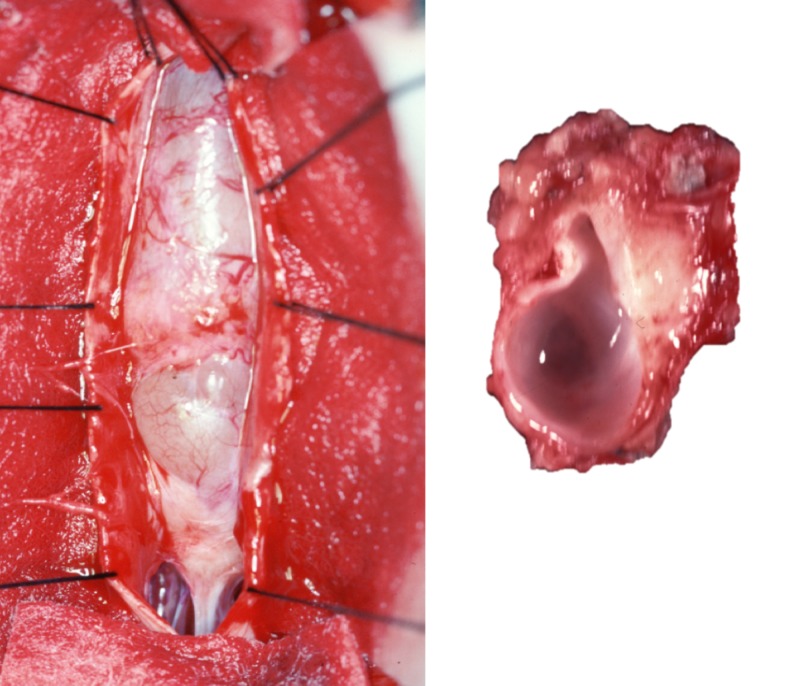
Left: Intraoperative view of a spinal NEC with dura mater tented laterally with sutures; Right: gross specimens of NEC transected sagittally revealing the smooth inner lining of the cyst.

Total removal, subtotal resection, fenestration of cyst, and simple aspiration are the surgical options [4,10]. Although total removal is the optimal treatment approach for its lowest recurrence rate, the ventral predilection site of the lesions or their close adherence to the spinal cord may not permit the complete excision (Figure 5) [3]. Under these circumstances, incomplete microdissection, instead, is offered. However, this treatment option is associated with a higher recurrence rate, necessitating further management [10-11]. Cystic fluid, if leaked into the intramedullary cavity, may cause meningeal irritation, and this is more prevalent in newborns and infants [11]. Aspiration is used in combination with resection to remove the cystic content prior to the excision of the wall and to relieve the local mass effect temporarily [4]. Aspiration monotherapy, however, is not practiced as it does not offer a long-term therapeutic effect [10]. Fenestration, which consists of establishing a communication between the cystic lumen and the subarachnoid space, is another proposed method to manage NEC. The shunting mechanism alleviates the growth of the non-communicating cystic mass [21]. This method is also offered in conjunction with a subtotal excision of a cyst to prevent the return of clinical manifestations [3-4,6,8,10-11,21,26].

## Conclusions

Electron microscopy, histological markers, special stains, and immunochemistry are tools to confirm the diagnosis of NEC. The majority of NECs are located anterior to the spinal cord. Although the proposed mechanisms for NEC do not clearly explain all forms of these entities, molecular backgrounds have been established. The complete resection of the cyst remains the best surgical management with the lowest incidence of recurrence.

## References

[1] Pavon V, Pratico AD, Caltabiano R (2017). Cervical neurenteric cyst and Klippel-Feil syndrome: an abrupt onset of myelopathic sign in a young patient. J Pediatr Surg Case Rep.

[2] Fortuna A, Mercuri S (1983). Intradural spinal cysts. Acta Neurochir (Wien).

[3] Kim HJ, Min KS, Kim YG, Kim DH (2018). Cervical intramedullary neurenteric cyst in an elderly patient. J Korean Neurosurg Soc.

[4] Cai C, Shen C, Yang W, Zhang Q, Hu X (2008). Intraspinal neurenteric cysts in children. Can J Neurol Sci.

[5] Vachhani JA, Fassett DR (2012). Intramedullary neurenteric cyst associated with a tethered spinal cord: case report and literature review. Surg Neurol Int.

[6] Rauzzino MJ, Tubbs RS, Alexander E, Grabb PA, Oakes WJ (2018). Spinal neurenteric cysts and their relation to more common aspects of occult spinal dysraphism. Neurosurg Focus.

[7] Wilkins RH, Rossitch JR (1995). Intraspinal cysts. Disorders of the Pediatric Spine.

[8] Kida K, Tani T, Kawazoe T, Hiroi M (2018). A recurrent cervical neurenteric cyst treated anteriorly: safe, gross-total excision facilitated by prophylactic unilateral vertebral artery exposure, microdissection, and spinal cord monitoring - a case report and technical note. Case Rep Ortho.

[9] Ziu M, Vibhute P, Vecil GG, Henry J (2010). Isolated spinal neurenteric cyst presenting as intramedullary calcified cystic mass on imaging studies: case report and review of literature. Neuroradiology.

[10] Takase T, Ishigawa M, Nishi S (2003). A recurrent intradural cervical neurenteric cyst operated on using an anterior approach. Surg Neurol.

[11] De Oliveira RS, Cinalli G, Roujeau T, Sainte-Rose C, Pierre-Kahn A, Zerah M (2018). Neurenteric cysts in children: 16 consecutive cases and review of the literature. J Neurosurg.

[12] Rhaney K, Barclay GPT (1959). Enterogenous cysts and congenital diverticula of the alimentary canal with abnormalities of the vertebral column and spinal cord. J Pathol Bacteriol.

[13] Macdonald RL, Schwartz ML, Lewis AJ (1991). Neurenteric cyst located dorsal to the cervical spine: case report. Neurosurgery.

[14] Beardmore HE, Wiglesworth FW (1958). Vertebral anomalies and alimentary duplications: clinical and embryological aspects. Pediatr Clin of N Am.

[15] Preece MT, Osborn AG, Chin SS, Smirniotopoulos JG (2018). Intracranial neurenteric cysts: imaging and pathology spectrum. Am J Neuroradiol.

[16] Wilkens RH, Odom GL (1976). Spinal intradural cysts. in tumors of the spine and spinal cord, part II. Handbook of Clinical Neurology.

[17] Miller CM, Wang BH, Moon SJ, 3 Chen E, Wang H (2014). Neurenteric cyst of the area postrema. Case Rep Neurol Med.

[18] Park CH, Hyun SJ, Kim KJ, Kim HJ (2012). Spinal intramedullary ependymal cysts: a case report and review of the literature. J Korean Neurosurg Soc.

[19] Nagano S, Ijiri K, Kawabata R, Zenmyo M, Yone K, Kitajima S, Komiya S (2010). Ependymal cyst in the conus medullaris. Clin Neurosci.

[20] Lee CW, Yoon SM, Kim YJ, Yun IG (2018). Endodermal cyst of the posterior fossa. J Korean Neurosurg Soc.

[21] Kapoor V, Johnson DR, Fukui MB, Rothfus WE, Jho HD (2018). Neuroradiologic-pathologic correlation in a neurenteric cyst of the clivus. Am J Neuroradiol.

[22] Goes P, Vaz-Guimaraes F, Suriano IC, Araujo S, Zymberg ST (2018). Supratentorial neurenteric cyst: analysis of 45 cases in the literature. Interdiscip Neurosurg.

[23] Chen CT, Lai HY, Jung SM, Lee CY, Wu CT, Lee ST (2016). Neurenteric cyst or neuroendodermal cyst? Immunohistochemical study and pathogenesis. World Neurosurg.

[24] Priamo FAI, Jimenez ED, Benardete EA (2011). Posterior fossa neurenteric cysts can expand rapidly: case report. Skull Base Rep.

[25] Yamamoto J, Shimajiri S, Akiba D, Nakano Y, Nishizawa S (2017). Intracranial neurenteric cyst with an enhanced mural nodule and melanin pigmentation: radiologic-pathologic correlation. World Neurosurg.

[26] Ergün R, Akdemir G, Gezici AR, Kara C, Ergüngör F (2000). Craniocervical neurenteric cyst without associated abnormalities. Pediatr Neurosurg.

